# Supplementary material for: Dendritic Cells Pulsed with HAM/TSP Exosomes Sensitize CD4 T Cells to Enhance HTLV-1 Infection, Induce Helper T-Cell Polarization, and Decrease Cytotoxic T-Cell Response
Source: Viruses. 2024 Sep 10;16(9):1443. doi: 10.3390/v16091443 (PMC11436225; doi:10.3390/v16091443)
Supplement: Supplementary file 1 [file viruses-16-01443-s001.zip › viruses-3185238-Revision-Supplemental/viruses-3185238-Revision-supplemental .pptx]

## Slide 1
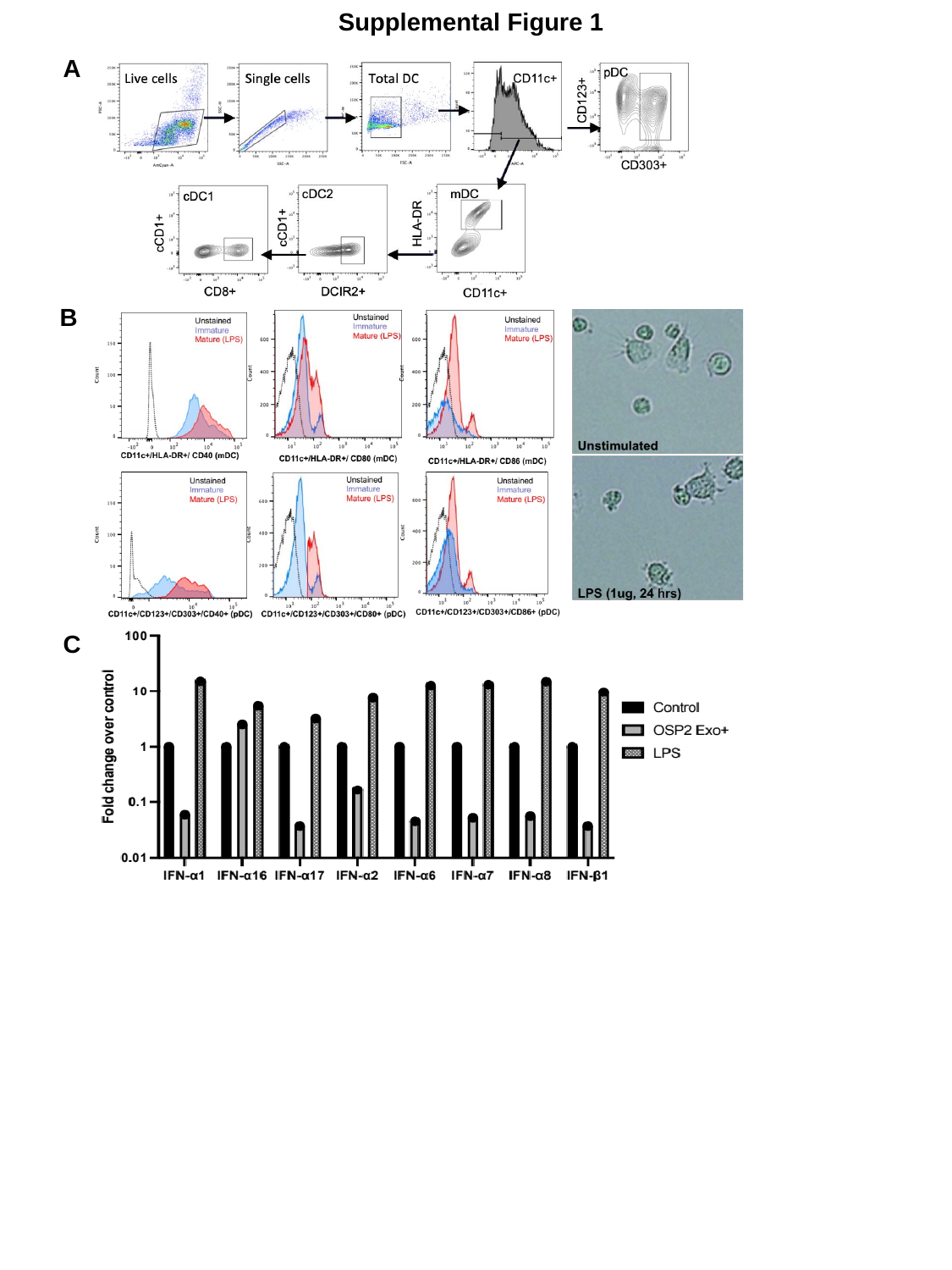

Supplemental Figure 1
A
B
C

## Slide 2
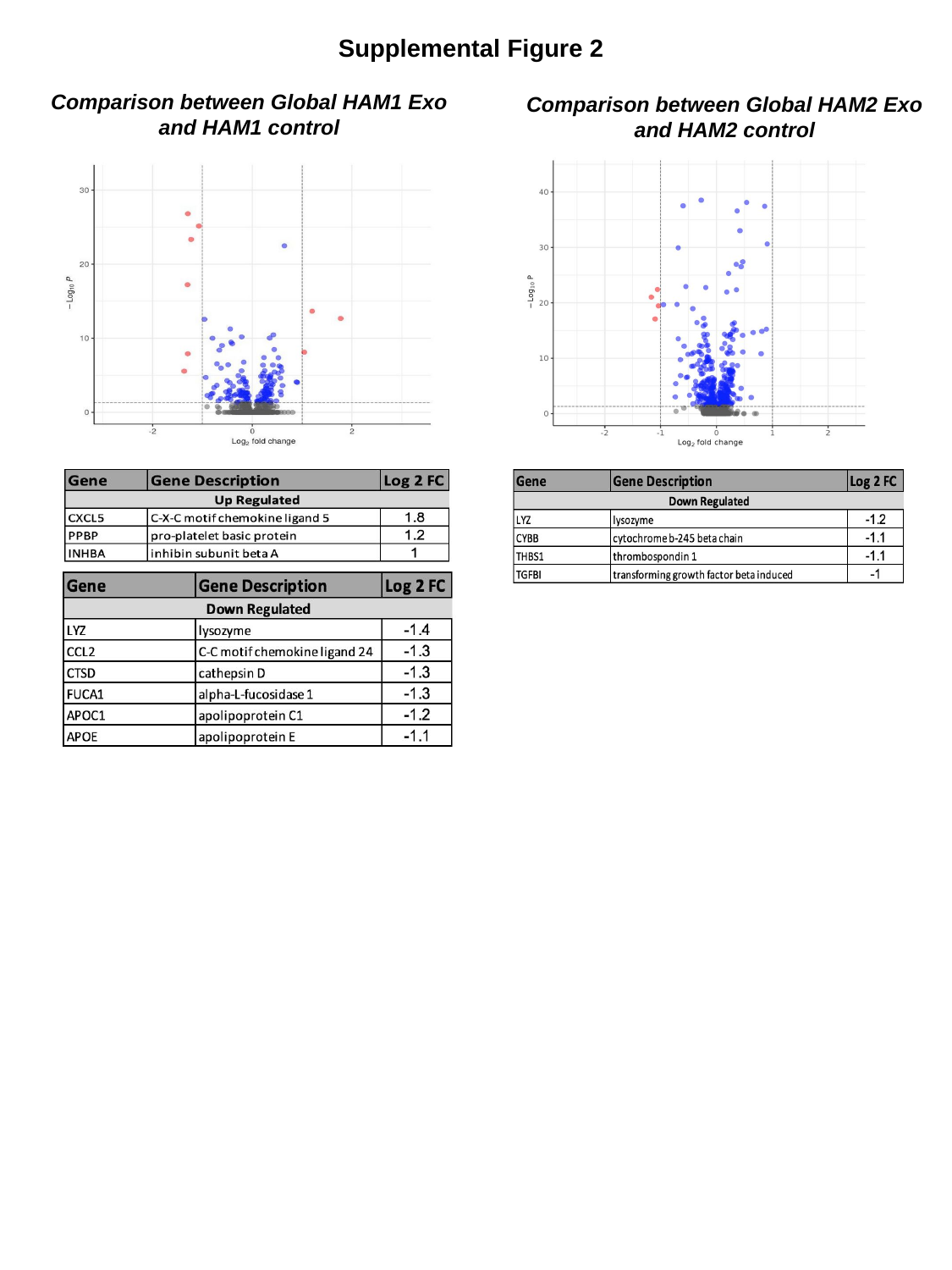

Supplemental Figure 2
Comparison between Global HAM2 Exo and HAM2 control
Comparison between Global HAM1 Exo and HAM1 control

## Slide 3
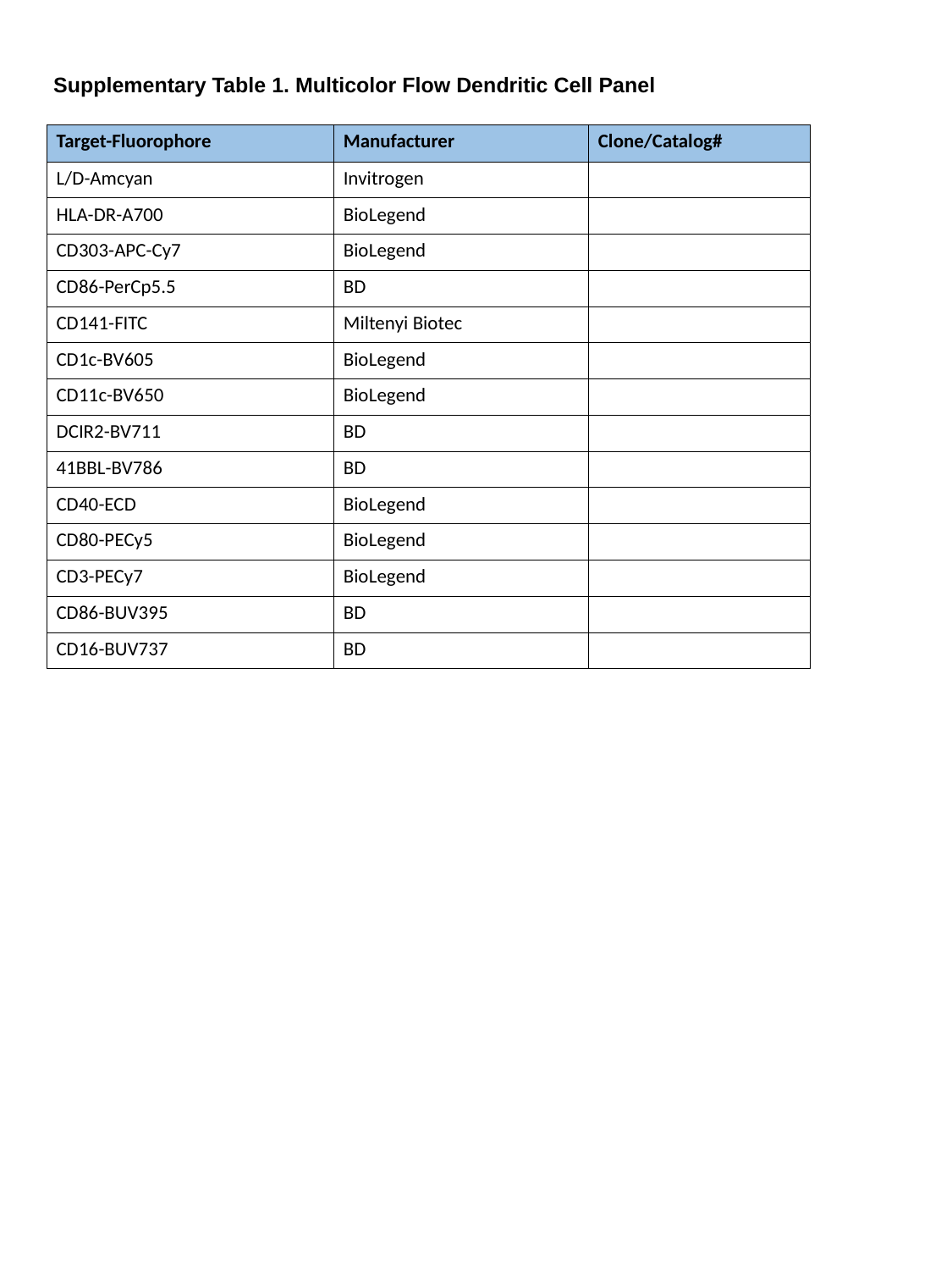

Supplementary Table 1. Multicolor Flow Dendritic Cell Panel
| Target-Fluorophore | Manufacturer | Clone/Catalog# |
| --- | --- | --- |
| L/D-Amcyan | Invitrogen | |
| HLA-DR-A700 | BioLegend | |
| CD303-APC-Cy7 | BioLegend | |
| CD86-PerCp5.5 | BD | |
| CD141-FITC | Miltenyi Biotec | |
| CD1c-BV605 | BioLegend | |
| CD11c-BV650 | BioLegend | |
| DCIR2-BV711 | BD | |
| 41BBL-BV786 | BD | |
| CD40-ECD | BioLegend | |
| CD80-PECy5 | BioLegend | |
| CD3-PECy7 | BioLegend | |
| CD86-BUV395 | BD | |
| CD16-BUV737 | BD | |

## Slide 4
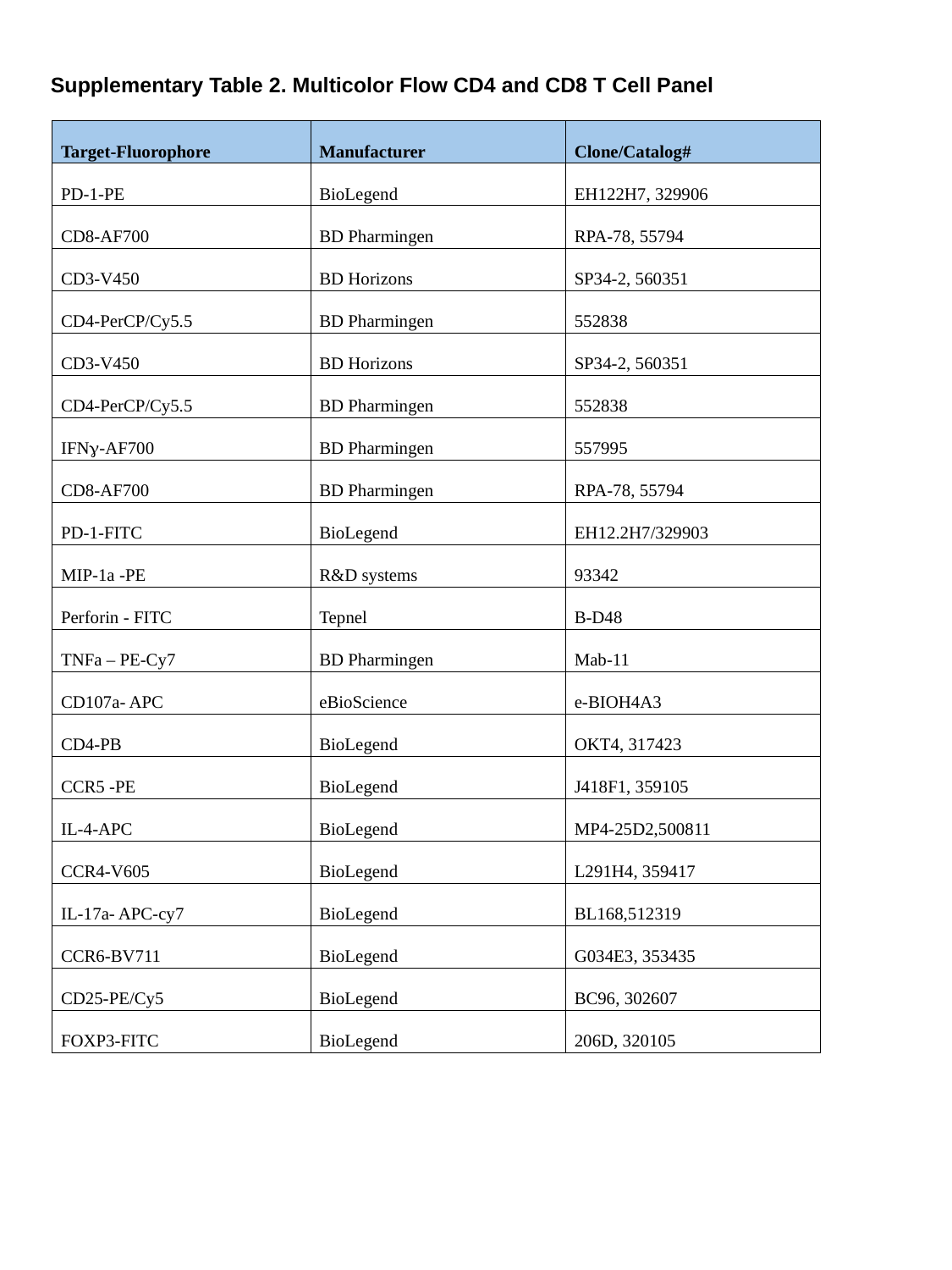

Supplementary Table 2. Multicolor Flow CD4 and CD8 T Cell Panel
| Target-Fluorophore | Manufacturer | Clone/Catalog# |
| --- | --- | --- |
| PD-1-PE | BioLegend | EH122H7, 329906 |
| CD8-AF700 | BD Pharmingen | RPA-78, 55794 |
| CD3-V450 | BD Horizons | SP34-2, 560351 |
| CD4-PerCP/Cy5.5 | BD Pharmingen | 552838 |
| CD3-V450 | BD Horizons | SP34-2, 560351 |
| CD4-PerCP/Cy5.5 | BD Pharmingen | 552838 |
| IFNγ-AF700 | BD Pharmingen | 557995 |
| CD8-AF700 | BD Pharmingen | RPA-78, 55794 |
| PD-1-FITC | BioLegend | EH12.2H7/329903 |
| MIP-1a -PE | R&D systems | 93342 |
| Perforin - FITC | Tepnel | B-D48 |
| TNFa – PE-Cy7 | BD Pharmingen | Mab-11 |
| CD107a- APC | eBioScience | e-BIOH4A3 |
| CD4-PB | BioLegend | OKT4, 317423 |
| CCR5 -PE | BioLegend | J418F1, 359105 |
| IL-4-APC | BioLegend | MP4-25D2,500811 |
| CCR4-V605 | BioLegend | L291H4, 359417 |
| IL-17a- APC-cy7 | BioLegend | BL168,512319 |
| CCR6-BV711 | BioLegend | G034E3, 353435 |
| CD25-PE/Cy5 | BioLegend | BC96, 302607 |
| FOXP3-FITC | BioLegend | 206D, 320105 |

## Slide 5
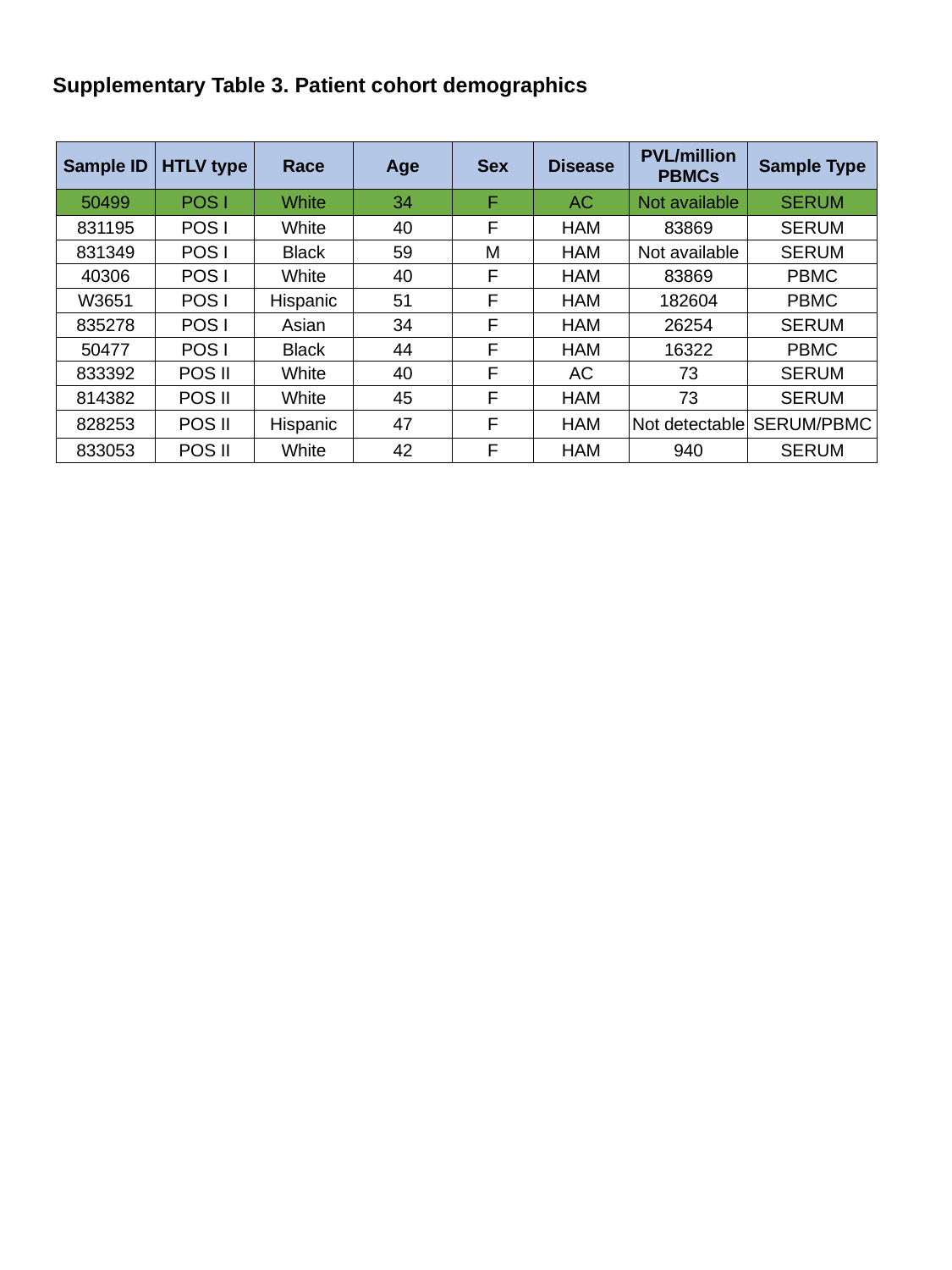

Supplementary Table 3. Patient cohort demographics
| Sample ID | HTLV type | Race | Age | Sex | Disease | PVL/million PBMCs | Sample Type |
| --- | --- | --- | --- | --- | --- | --- | --- |
| 50499 | POS I | White | 34 | F | AC | Not available | SERUM |
| 831195 | POS I | White | 40 | F | HAM | 83869 | SERUM |
| 831349 | POS I | Black | 59 | M | HAM | Not available | SERUM |
| 40306 | POS I | White | 40 | F | HAM | 83869 | PBMC |
| W3651 | POS I | Hispanic | 51 | F | HAM | 182604 | PBMC |
| 835278 | POS I | Asian | 34 | F | HAM | 26254 | SERUM |
| 50477 | POS I | Black | 44 | F | HAM | 16322 | PBMC |
| 833392 | POS II | White | 40 | F | AC | 73 | SERUM |
| 814382 | POS II | White | 45 | F | HAM | 73 | SERUM |
| 828253 | POS II | Hispanic | 47 | F | HAM | Not detectable | SERUM/PBMC |
| 833053 | POS II | White | 42 | F | HAM | 940 | SERUM |
