# Supplementary figures and images for: Dendritic Cells Pulsed with HAM/TSP Exosomes Sensitize CD4 T Cells to Enhance HTLV-1 Infection, Induce Helper T-Cell Polarization, and Decrease Cytotoxic T-Cell Response
Source: Viruses. 2024 Sep 10;16(9):1443. doi: 10.3390/v16091443 (PMC11436225; doi:10.3390/v16091443)

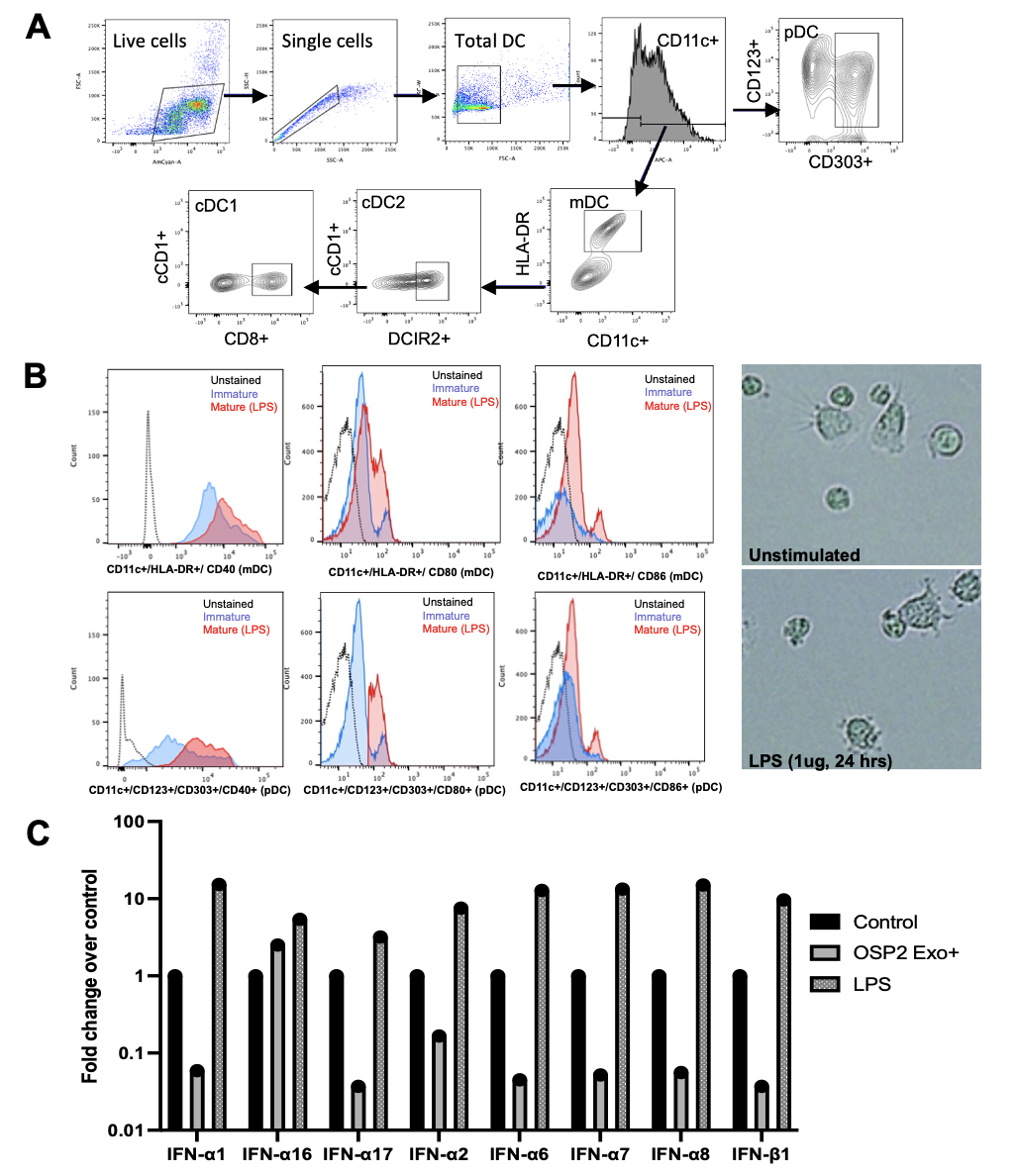

Supplement: Supplementary file 1 [file viruses-16-01443-s001.zip › viruses-3185238-Revision-Supplemental/viruses-3185238-Revision-Supplemental Figure 1.jpg]
